# Supplementary material for: Acidocin A and Acidocin 8912 Belong to a Distinct Subfamily of Class II Bacteriocins with a Broad Spectrum of Antimicrobial Activity
Source: Int J Mol Sci. 2024 Sep 19;25(18):10059. doi: 10.3390/ijms251810059 (PMC11432624; doi:10.3390/ijms251810059)
Supplement: Supplementary file 1 [file ijms-25-10059-s001.zip › ijms-3161533-supplementary.pdf]

Supplementary information for

Acidocin A and Acidocin 8912 Belong to a Distinct # Subfamily of Class II Bacteriocins with a Broad Spectrum # of Antimicrobial Activity

Daria V. Antoshina [1,†], Sergey V. Balandin [1,*,†], Ekaterina I. Finkina [1], Ivan V. Bogdanov [1], Sofia I. Eremchuk [1], # Daria V. Kononova [1,2], Alena A. Kovrizhnykh [1,2] and Tatiana V. Ovchinnikova [1,2,3]

[1]# M.M. Shemyakin and Yu.A. Ovchinnikov Institute of Bioorganic Chemistry, Russian Academy of Sciences, 117997 Moscow, Russia; riruka11@mail.ru (D.V.A.); finkina@mail.ru (E.I.F.); contraton@mail.ru (I.V.B.); sofiia.eremchuk@gmail.com (S.I.E.); kononova.dv@phystech.edu (D.V.K.); potemkina.aa@phystech.edu (A.A.K.); ovch@ibch.ru (T.V.O.)

[2]# Moscow Center for Advanced Studies, 123592 Moscow, Russia

[3]# Department of Biotechnology, I.M. Sechenov First Moscow State Medical University, 119991 Moscow, Russia

* Correspondence: arenicin@mail.ru; Tel.: +7-495-335-0900

† These authors contributed equally to this work.

Table S1. The recombinant peptides obtained in this work.

Peptide	**Final yield of recombinant peptide, mg/L**	**Hydrophobicity index ***	**Retention time during RP-HPLC, min**	**Calculated $[M+H]^+$ monoisotopic mass, Da ***	**Experimental $[M+H]^+$ monoisotopic mass, Da**
Avicin A	5.4	–0.188	35.8	4288.7	4289.3
Acidocin A	6.2	–0.298	47.2	6500.6	6500.2
AcdA(10–48)	4.1	–0.236	28.5	4497.6	4496.7
AcdA(10–39)	3.2	–0.430	22.4	3431.0	3430.6
AcdA(10–33)	4.4	–0.475	29.0	2764.3	2764.1
AcdA(10–33,C31S)	2.6	–0.613	29.1	2748.0	2748.2
AcdA(32–58)	4.8	–0.045	47.8	4474.6	4474.9
AcdA(19–58)	3.9	–0.233	42.1	3052.7	3053.4
AcdA(1–31)	5.1	–0.355	23.8	3463.8	3462.5
AviA(1–19)-AcdA(32–58)	1.2	–0.233	46.2	6349.4	6350.0
AviA(1–19)-AcdA(19–58)	3.4	–0.269	46.5	4295.2	4294.9
Acidocin 8912	2.7	–1.012	41.5	3217.6	3218.1
Acidocin 8912A	1.3	–0.546	45.1	7555.1	7555.6

* Expasy ProtParam tool (https://web.expasy.org/protparam) was used to calculate the Kyte-Doolittle grand average hydropathicity indexes (GRAVY) and $[M+H]^+$ monoisotopic masses of recombinant peptides. The maximum and minimum values of hydrophobicity index are +4.5 for Ile and −4.5 for Arg. $[M+H]^+$ monoisotopic masses were calculated taking into account the presence of two Cys residues forming a disulfide bond in avicin A, acidocin A, AcdA(10–48), AcdA(10–39), AcdA(10–33), AcdA(1–31), and AviA-AcdA hybrids. Experimental $[M+H]^+$ monoisotopic masses were determined using MALDI-TOF mass spectrometry.

A

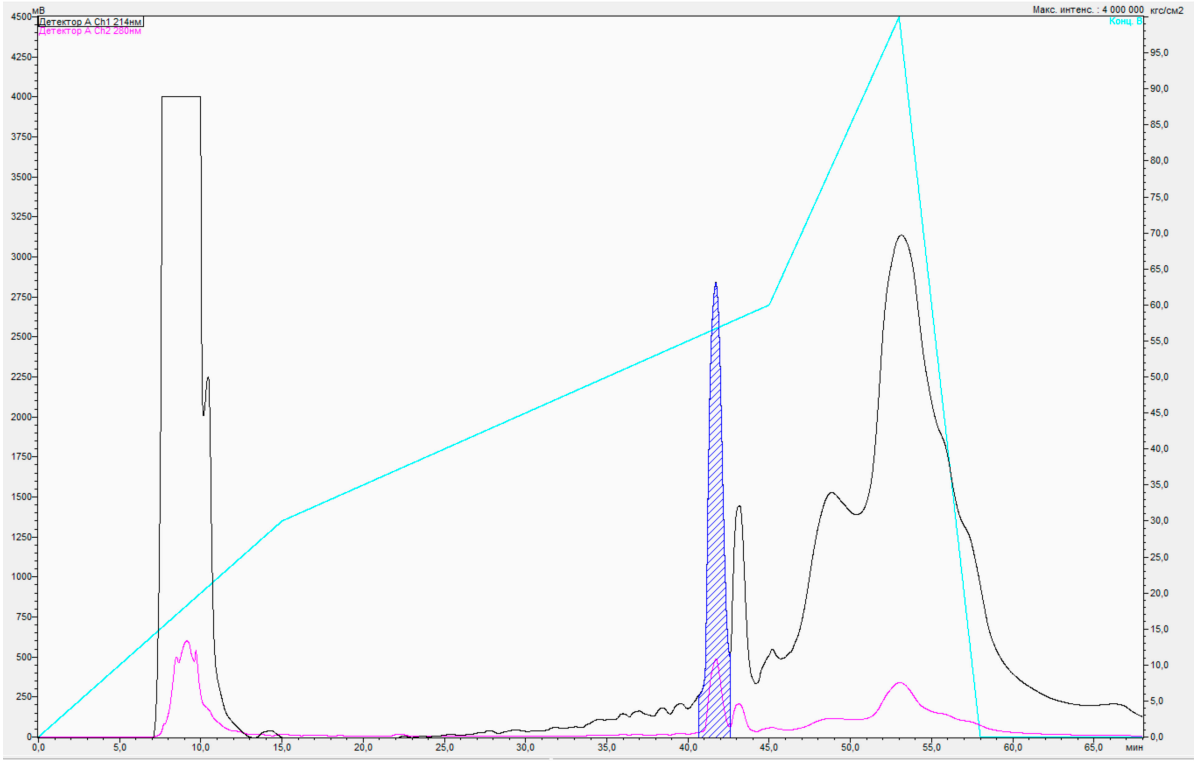

B

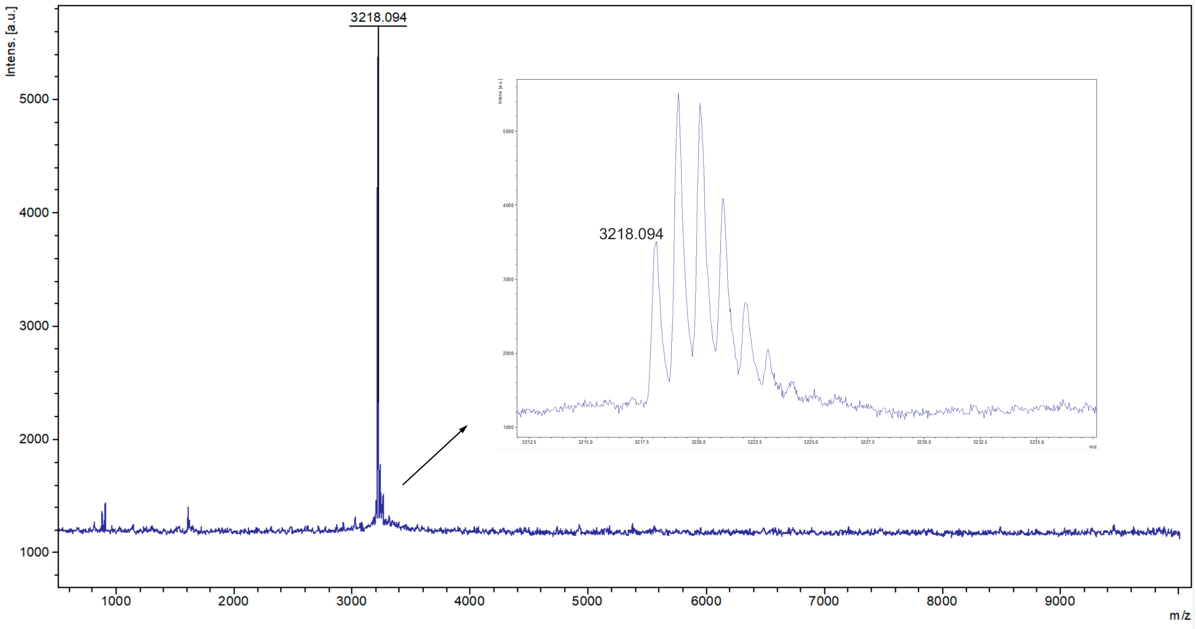

Figure S1. (A) Reversed-phase high-performance liquid chromatography (RP-HPLC) purification of the recombinant acidocin 8912 and **(B)** MALDI-TOF mass-spectrometry analysis of the collected fraction. RP-HPLC was performed with a gradient (cyan line) from 5 to 80% (v/v) of acetonitrile in water containing 0.1% TFA for 58 min. Peptide elution was detected spectrophotometrically at 214 and 280 nm (black and pink lines). The collected recombinant peptide fraction is marked with blue shading.

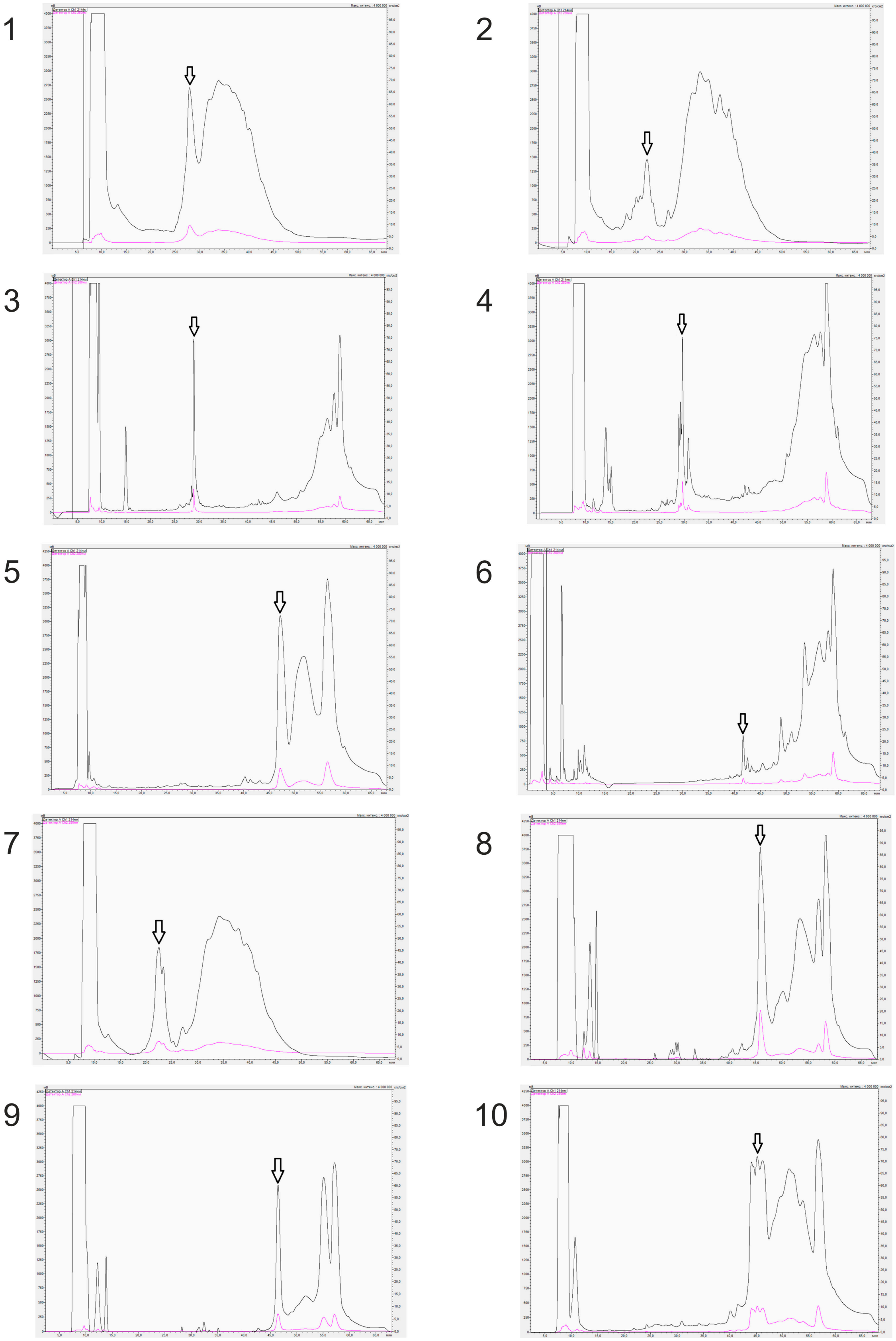

Figure S2. RP-HPLC purification of the recombinant bacteriocins: 1 — AcdA(10–48); 2 — AcdA(10–39); 3 — AcdA(10–33); 4 — AcdA(10–33,C31S); 5 — AcdA(32–58); 6 — AcdA(19–58); 7 — AcdA(1–31); 8 — AviA(1–19)-AcdA(32–58); 9 — AviA(1–19)-AcdA(19–58); 10 — Acidocin 8912A. The purification conditions were the same as those for acidocin 8912. The collected recombinant peptide fractions are marked with arrows.

Table S2. Bacterial strains used in this study.

**Bacterial strain**	**Characteristics (source, antibiotic resistance, etc.)**
**Gram-positive bacteria**	
Bacillus licheniformis B-511	All-Russian Collection of Microorganisms (VKM)
Bacillus subtilis B-886	All-Russian Collection of Microorganisms (VKM)
Bacillus subtilis B-2895	All-Russian Collection of Industrial Microorganisms (VKPM)
Enterococcus faecium E19	Clinical isolate, vancomycin-resistant, Collection of Institute of Experimental Medicine (FSBSI "IEM")
Enterococcus faecium E62	Clinical isolate, vancomycin-resistant, Collection of Institute of Experimental Medicine (FSBSI "IEM")
Enterococcus faecium E63	Clinical isolate, vancomycin-resistant, Collection of Institute of Experimental Medicine (FSBSI "IEM")
Lactococcus lactis ssp. *lactis* MK43	Laboratory strain
Lactococcus lactis ssp. *lactis* bv. *diacetylactis* MK66	Laboratory strain
Lactococcus cremoris B-1569	All-Russian Collection of Industrial Microorganisms (VKPM)
Listeria monocytogenes EGD	Collection of Institute of Experimental Medicine (FSBSI "IEM")
Micrococcus luteus Ac-2229	All-Russian Collection of Microorganisms (VKM)
Mycobacterium phlei Ac-1291	All-Russian Collection of Microorganisms (VKM)
Mycobacterium smegmatis MC2 155	Collection of Laboratory of regulatory transcriptomics (IBCH RAS)
Staphylococcus aureus 209P	Laboratory strain (ATCC collection)
**Gram-negative bacteria**	
Acinetobacter baumannii (XDR CI 2675)	Extensively drug resistant clinical isolate* (MBL+)
Escherichia coli ML-35p	Laboratory strain (ATCC collection)
Escherichia coli SQ110	Laboratory strain (provided by prof. A.S. Mankin)
Escherichia coli (XDR CI 1057)	Clinical isolate, extensively drug resistant (urine, urinary tract infection; ESBL+)
Escherichia coli ATCC 25922	Laboratory strain (ATCC collection)
Escherichia coli CI 214	Clinical isolate (urine, acute pyelonephritis)
Escherichia coli SBS 1936	Laboratory strain (IMG RAS collection)
Pseudomonas aeruginosa PAO1	Laboratory strain (ATCC collection)

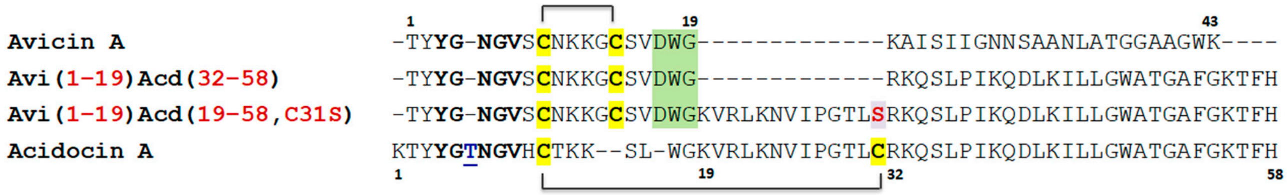

Figure S3. The structure of avicin A - acidocin A hybrids. Cys residues are highlighted in yellow; the supposed hinge region in avicin A is highlighted with green; amino acid residues included in pediocin box are shown in bold; the non-canonical threonine residue is underlined.

Table S3. Antibacterial activity of avicin A — acidocin A hybrid peptides compared to original acidocin A (AcdA) and avicin A (AviA).

**Bacterial Strains**	**Minimum Inhibitory Concentration (μM)**			
	**AcdA**	**AviA(1–19) -AcdA(32–58)**	**AviA(1–19) -AcdA(19–58)**	**AviA**
Listeria monocytogenes EGD	>32	>32	>32	<0.125
Lactococcus lactis ssp. *lactis* bv. *diacetylactis* MK66	0.5	16	1	>32
Lactococcus lactis ssp. *lactis* MK43	1	32	nd	>32
Lactococcus cremoris B-1589	4	>32	8	>32
Bacillus subtilis B-886	2	>32	16	>32
Bacillus subtilis B-2895	4	32	16	>32
Bacillus licheniformis B-511	2	>32	>32	>32
Mycobacterium phlei Ac-1221	2	>32	16	>32
Micrococcus luteus Ac-2229	8	>32	>32	>32
E. coli SQ110	2	16	8	>32
E. coli ML-35p	2	>32	32	>32
E. coli ATCC 25922	4	>32	16	>32
E. coli XDR CI 1057	8	>32	>32	>32

Abbreviation: nd — not determined.

Table S4. Fungal strains used in this study.

**Fungal strain**	**Characteristics (source, antibiotic resistance)**
C. *albicans* ATCC 18804	Laboratory strain (ATCC collection)
C. *albicans* ATCC 10231	Laboratory strain (ATCC collection), azole- and anidulafungin-resistant
C. *albicans* v47a	Clinical isolate from patient with HIV infection (provided by G.N. Gabrichevsky Research Institute for Epidemiology and Microbiology, Moscow, Russia)
C. *albicans* 9.1	Clinical isolate from patient with HIV infection (provided by G.N. Gabrichevsky Research Institute for Epidemiology and Microbiology, Moscow, Russia), azole-resistant
C. tropicalis v13a4/2	Clinical isolate from patient with HIV infection (provided by G.N. Gabrichevsky Research Institute for Epidemiology and Microbiology, Moscow, Russia)
C. krusei 225/2	Clinical isolate from patient with HIV infection (provided by G.N. Gabrichevsky Research Institute for Epidemiology and Microbiology, Moscow, Russia)
C. glabrata 252/2	Clinical isolate from patient with HIV infection (provided by G.N. Gabrichevsky Research Institute for Epidemiology and Microbiology, Moscow, Russia)

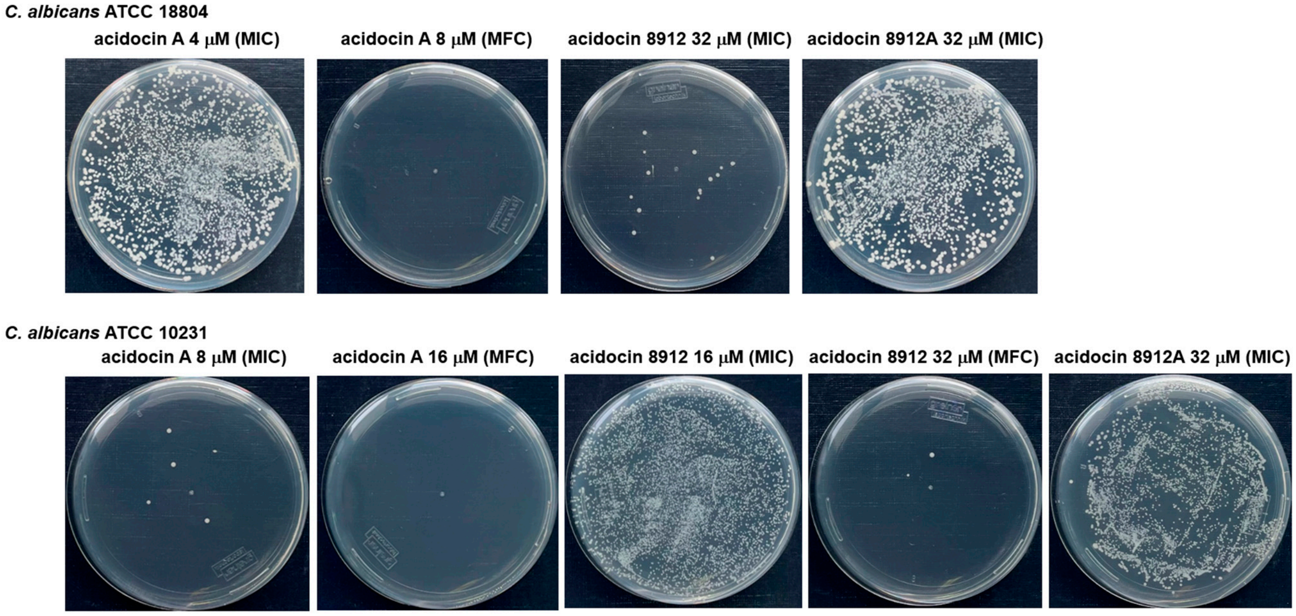

Figure S4. Determination of MFC by plating the contents of the wells with peptide concentrations corresponding to MICs and above onto a solid growth medium and incubating the plates for 24 hours.

Table S5. Effect of salts and fetal bovine serum (FBS) on the activity of peptides against *C. albicans* ATCC 18804 (concentrations in μM).

Peptide	No salts, no FBS		Na^+		Ca^{2+}		Mg^{2+}		FBS	
	MIC	MFC	MIC	MFC	MIC	MFC	MIC	MFC	MIC	MFC
Acidocin A	4	8	>16	nd	>16	nd	4	8	>16	nd
Acidocin 8912	32	>32	>32	nd	>32	nd	>32	nd	>32	nd
Acidocin 8912A	32	>32	>32	nd	>32	nd	32	>32	>32	nd
Avicin A	>32	nd	>32	nd	>32	nd	>32	nd	>32	nd

Abbreviation: nd — not determined.

Table S6. Sensitivity of the clinical isolates of *Candida albicans* to conventional antimycotics (according to [S1]).

Antifungal agent	MICs (mg/L)*	
	v47a3	9.1
Amphotericin B	0.5	1
Anidulafungin	0.03	0.06
Fluconazole	8	256
Flucytosine	≤0.06	≤0.06
Itraconazole	0.12	16
Micafungin	0.008	0.015
Posaconazole	0.12	8
Voriconazole	0.5	8
Caspofungin	0.06	0.12

*MIC – minimal inhibitory concentration (>90% inhibition of fungal growth for amphotericin B, but 50% growth inhibition for other compounds).

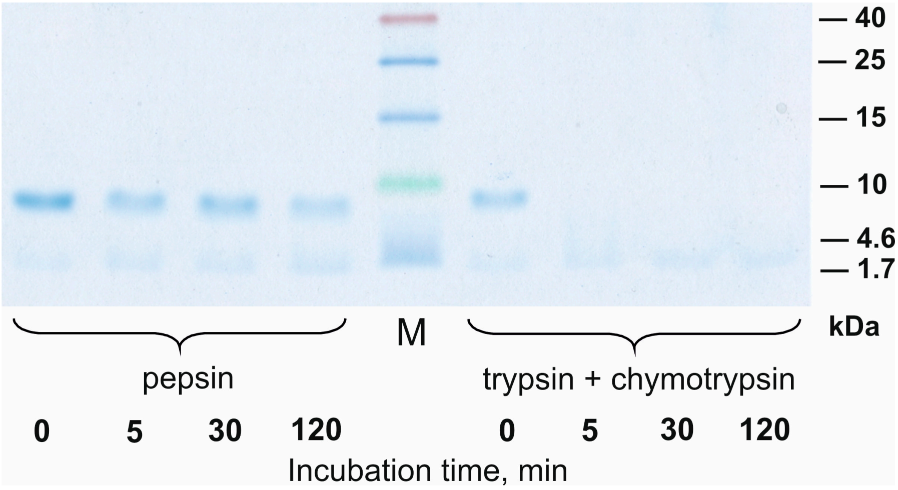

Figure S5. Resistance of acidocin A to digestion by the major proteases of the gastroenteric tract. Spectra Multicolor Low Range Protein Ladder (Thermo Scientific, # 26628) was used as the molecular weight standard.

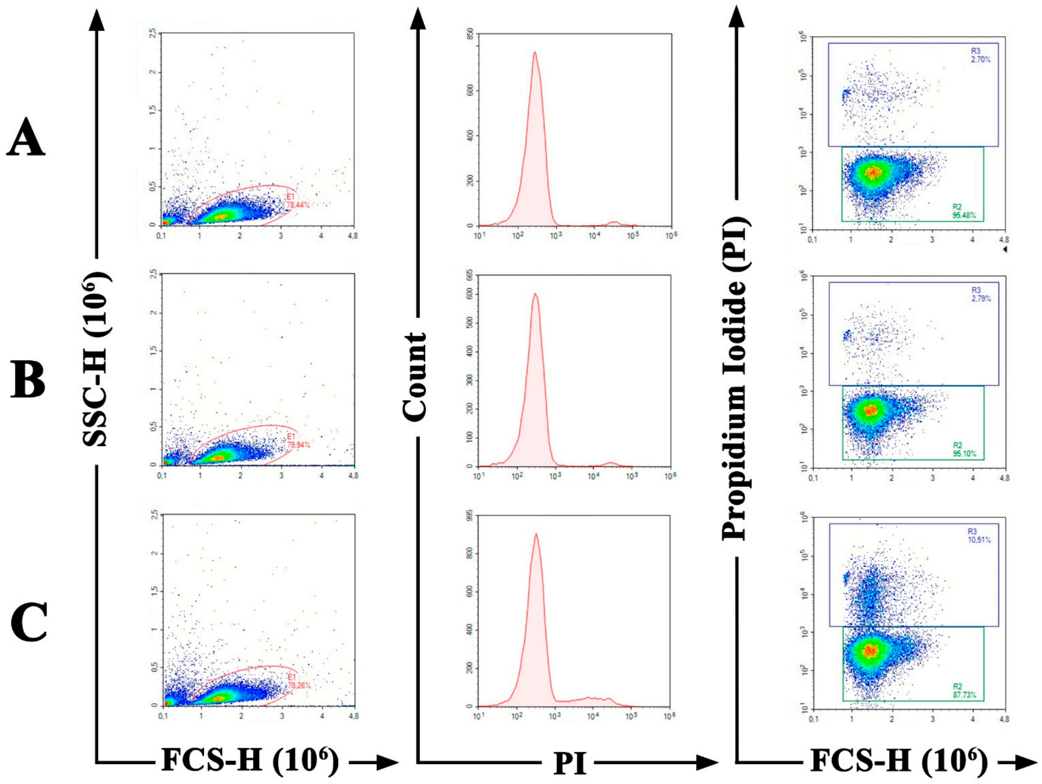

Figure S6. The effect of amphotericin B at concentrations of 0.5 × MIC (A), MIC (B) and 4 × MIC (C) on the cell membrane permeability of *C. albicans* ATCC 18804, measured by PI uptake. Left panel: The side scatter (SSC-H) versus the forward scatter (FCS-H) shows morphological homogeneity of the cell population. Middle panel demonstrates PI uptake versus count. Right panel: PI plot versus the forward scatter (FCS-H) shows the shift of population of PI-positive cells along the y-axis.

References

S1. UCAST Definitive document E.DEF 7.3.2 Method for the determination of broth dilution minimum inhibitory concentrations of antifungal agents for yeasts. https://www.eucast.org/astoffungi/methodsinantifungalsusceptibilitytesting/susceptibility_testing_of_yeasts/
